# The state of the art in the analysis of two-dimensional gel electrophoresis images

**DOI:** 10.1007/s00253-007-1128-0

**Published:** 2007-08-23

**Authors:** Matthias Berth, Frank Michael Moser, Markus Kolbe, Jörg Bernhardt

**Affiliations:** 1 DECODON GmbH, Rathenau-Strasse 49a, 17489 Greifswald, Germany; 2 Institute of Microbiology, Greifswald University, Jahnstrasse 15, 17487 Greifswald, Germany

**Keywords:** 2-D gel electrophoresis, Image analysis, Proteome maps, Warping, Statistics

## Abstract

Software-based image analysis is a crucial step in the biological interpretation of two-dimensional gel electrophoresis experiments. Recent significant advances in image processing methods combined with powerful computing hardware have enabled the routine analysis of large experiments. We cover the process starting with the imaging of 2-D gels, quantitation of spots, creation of expression profiles to statistical expression analysis followed by the presentation of results. Challenges for analysis software as well as good practices are highlighted. We emphasize image warping and related methods that are able to overcome the difficulties that are due to varying migration positions of spots between gels. Spot detection, quantitation, normalization, and the creation of expression profiles are described in detail. The recent development of consensus spot patterns and complete expression profiles enables one to take full advantage of statistical methods for expression analysis that are well established for the analysis of DNA microarray experiments. We close with an overview of visualization and presentation methods (proteome maps) and current challenges in the field.

## Introduction

The last decade in life sciences was deeply influenced by the development of the “Omics” technologies (genomics, transcriptomics, proteomics, and metabolomics), which aim for a global view on biological systems. With these tools at hand, the scientific community is striving to build functional models to develop a global understanding of the living cell.

The analysis of the proteome as the final level of gene expression started out with techniques based on 2-D gel electrophoresis (O’Farrell 1975; Klose 1975) and extended its reach with semi-gel-free and shot gun gel-free liquid chromatography–mass spectrometry (LC–MS)-based techniques in recent years.

A comprehensive evaluation of the relative merits and weaknesses of gel-based and gel-free methods is beyond the scope of our review. Studies that compare the performance on similar samples (Wolff et al. 2006, 2007) indicate that the methods are complementary, i.e., their analytical windows overlap, but each has an exclusive set of proteins or modifications that were not identified with the other. Quantitative analysis based on LC–MS techniques is still in an early stage when considering available software and algorithms. Here, we focus on the computerized analysis of 2-D gels which are widely used in the scientific community. 2-D gels may separate up to 10,000 protein spots on one gel (Klose and Kobalz 1995). In a suitably equipped and experienced lab environment, 2-D gels are easy to handle, and they can be produced in a highly parallelized way. The software has meanwhile reached a level that allows for routine analysis of a large amount of samples with an investment of time that is much smaller than the efforts needed for the wet lab work.

The routine application of potent and easy-to-use software systems has been enabled by several improvements in 2-D gel image acquisition and analysis technology over the last decades (Aittokallio et al. 2005; Dowsey et al. 2003). Among these milestones, one should mention the introduction of the first computer-based analysis systems, still without a graphical user interface, in the late 1970s, among them ELSIE (Bossinger et al. 1979; Vo et al. 1981), GELLAB (Lemkin and Lipkin 1981a, b, c; Lemkin 1989), TYCHO (Anderson et al. 1981), HERMeS (Tarroux et al. 1989; Vincens and Tarroux 1987), GESA (Rowlands et al. 1988), and LIPS (Skolnick et al. 1982). This first generation of 2-D image analysis programs was followed by Elsie-4 (Olson and Miller 1988), Melanie (Appel et al. 1991; based on Elsie-4), and QUEST (Garrels 1989) since the mid-1980s. These programs used X-Windows-based graphical user interfaces on computer workstations. In the following years, the QUEST, TYCHO, and Melanie programs evolved to the commercially available PDQuest, Kepler, and Melanie III. While in the beginning such software needed exceptionally equipped workstations, in 1989, Nonlinear (Newcastle, UK) introduced Phoretix, the first 2-D gel analysis software running on desktop PCs. With the dropping hardware prices, the Melanie and PDQuest systems were also ported to PCs.

While until then none of the available systems gave visual feedback on the quality of spot matching, Melanie II introduced image adjustment based on a global polynomial transformation of the image’s geometry. This simplified the comparison of the raw images but only incompletely addressed the problem of positional variations caused by the electrophoretical separation process. Horgan et al. (1992) used for the first time a superimposing of false-colored 2-D images to simplify the finding of differences in spot patterns. Unlü et al. (1998) and Bernhardt et al. (1999) suggested different approaches that use superimposed and congruent false color images for the comparison of 2-D gels. This technology was improved by establishing positional correction by image warping of the raw 2-D gel images and commercialized with the first version of the Delta2D software from DECODON (Greifswald, Germany), coinciding with Compugen’s Z3 software in 2000 (Smilansky 2001). Both systems were able to completely remove distortions from the gel images and bring the spot patterns into congruency. See Table 1 for a list of current software.

**Table 1:** Current commercial software products for 2-D gel image analysis

Company	Products
Bio-Rad, Hercules, CA, USA, www.biorad.com	PDQuest, ProteomWeaver
Compugen, Tel Aviv, Israel, www.compugen.com	Z3 (discontinued)
DECODON, Greifswald, Germany, www.decodon.com	Delta2D
GE Healthcare, www.gelifesciences.com	Decyder 2D, ImageMaster Platinum*
Genebio, Geneva, Switzerland, www.genebio.com	*Melanie (ImageMaster Platinum)
Nonlinear Dynamics, Newcastle, UK, www.nonlinear.com	Progenesis, SameSpots
Syngene, Cambridge, UK, www.syngene.com	Dymension

With the ever-increasing capacity of available hardware, more advanced image processing methods became feasible. They use all of the available image information instead of condensing it to spot boundaries before processing. This simplifies image comparison and speeds up analysis dramatically but still produces expression profiles with gaps which significantly impede reliable gene expression analysis. The most recent milestone introduced by us in 2003 addressed this problem: An algorithm to combine the information of several gels into a so-called fusion gel makes it possible to generate a proteome map that is representative for the whole experiment (Luhn et al. 2003). On a proteome map, one can detect all spots of a whole experiment in a single gel image, whereas the average images proposed earlier suffer from dilution effects for weak and rare spots. The spots detected there can serve as a spot consensus pattern that is valid for the whole gel set of the experiment. The consensus spot pattern is then transferred according to the warping transform and used on all gels. This allows for 100% matching spots and, in turn, complete expression profiles for reliable statistical analysis (Voigt et al. 2006; Höper et al. 2006).

Depending on the workflow, one can put 2-D gel image analysis software into two broad categories:

1. Spot detection first: these are the classical packages where the image information is first condensed into a set of spot centers, boundaries, and possibly spot volumes for each image. Spot matching and subsequent creation of expression profiles are done based on the data about spot geometry and volumes.
2. Image warping first: these are packages where image warping is applied to remove running differences between gels, based on the whole image information. Spot detection is a separate and independent step. The creation of expression profiles is critically informed (and improved) by the data about positional differences between gels that were gained in the first step.

Historically, the “spot detection first” workflow was the only feasible way to proceed due to the limitations of available hardware. Software based on the second “image warping first” workflow, including Compugen’s Z3, DECODON’s Delta2D, and, most recently, Nonlinear’s SameSpots, is able to overcome many of the difficulties in spot matching that are hard to deal with when different migration positions add to the uncertainty of multiple separate spot detections. With the subsequent introduction of consensus spot patterns (in Delta2D 2003 and SameSpots 2006), one is able to virtually eliminate matching problems by using consistent spot patterns throughout the experiment. Recent comparisons between separate spot detection and using a consensus spot pattern (Eravci et al. 2007) show that the latter approach is able to find substantially more differentially expressed proteins, in a much shorter time. In other words, valuable information is lost due to spot matching problems that are inevitable when using the classical approach. We expect other vendors to evolve their products to use a workflow that is based on image warping and consensus spot patterns over the following years.

**Fig. 1:**
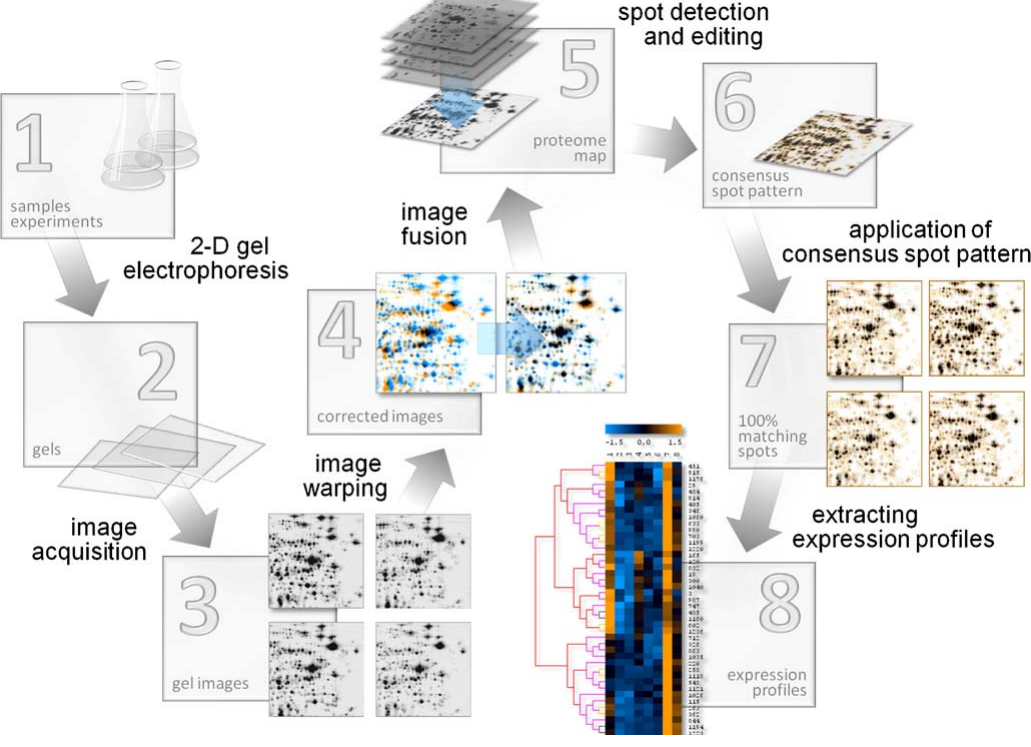
Analysis work flow of a 2-D-gel-based proteomics experiment in Delta2D. *1* Sample preparation; *2* 2-D gel electrophoresis; *3* 2-D gels are stained/detected and digitized; *4* spot positions are aligned across gel images by warping; *5* a proteome map/fusion gel image is generated by combining the images using a union fusion; *6* the union fusion image serves as basis for constructing the consensus spot pattern for the whole experiment; *7* the consensus spot pattern is transferred to all images and subsequently remodeled; *8* expression profiles are extracted and analyzed to find relevant proteins

**Fig. 2:**
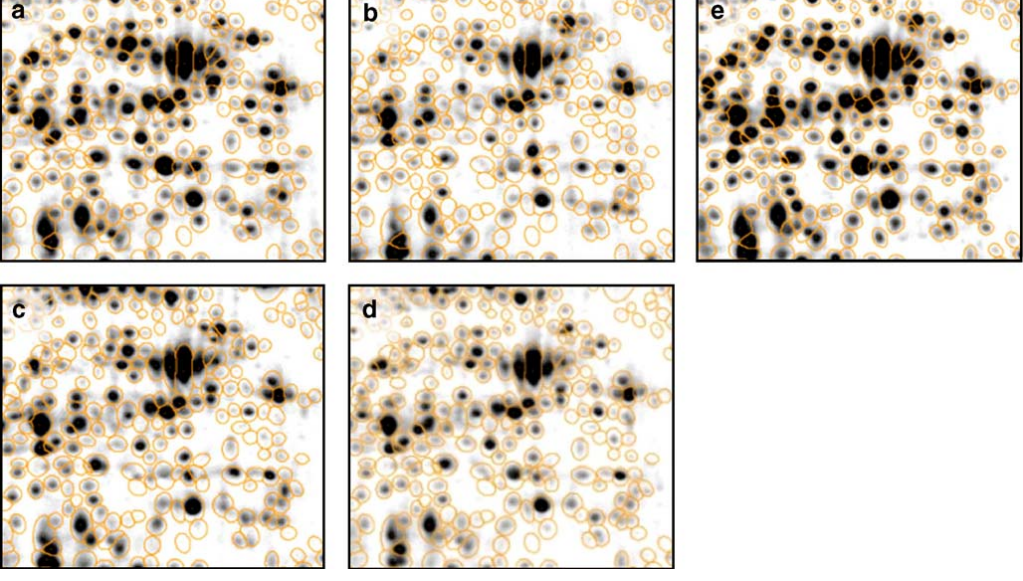
Consensus spot pattern applied to four gel images (**a**–**d**), before remodeling of spot shapes. The consensus spot pattern is generated by spot detection on the synthetic fusion image (**e**) which was computed from the original images

The typical workflow of a 2-D-gel-based proteomics analysis using the “image warping first” approach and consensus spot patterns can be described as follows (Fig. 1):

1. Performing a biological experiment or selecting a biological object of interest. The first sample preparation step is freezing the sample in the current state. This includes inactivation of all cellular processes that may change the proteome composition, preventing protease action, disintegration of the cell material, keeping or bringing the proteins into solution, removing or destroying macromolecules that may disturb the subsequent steps of the 2-D protocol (RNase and/or DNase treatment and centrifuging for cell debris removal). Alternatively or in combination with radiolabeling, covalent fluorescent labeling of proteins can be applied here.
2. Bringing the proteins into the gel and performing the 2-D separation by combining isoelectrofocussing in the first and sodium dodecyl sulfate (SDS) electrophoresis in the second dimension. An alternate 2-D approach uses the combination of two detergent treatments that resolve the protein molecules differently resulting in a scattered diagonal spot pattern (2D-16-BAC- or 2D-CTAB/SDS-polyacrylamide gel electrophoresis). A variety of staining techniques can be applied before or after separation to enable spot detection.
3. Capturing the gel images by using scanners, charge-coupled device (CCD) camera-based, or laser imaging devices. Depending on the protein labeling or staining techniques, a compatible imaging device has to be chosen. The capturing process results in one or more digitized computer images per gel that can be displayed with common image analysis software. The image capture step transforms the quantitative information of the gel into computer-readable data.
4. Correction of positional spot variations by image warping. 2-D electrophoresis results in spot patterns with variations in the spot positions between gels. Therefore, gel images are positionally corrected by a combination of global and local image transforms (image warping). The information about differences in spot positions that was gained in this step is reused later for image fusion and for the transfer of the consensus spot pattern.
5. Image fusion and proteome maps condense the image information of the whole experiment into one fusion image, also called a proteome map. The proteome map contains the information of all protein spots ever detected in the experiment.
6. Spot detection is performed on the proteome map. As a result, a consensus spot pattern is generated, which is valid for all gels in the experiment. It describes the position and the general shape of all protein spots from the experiment.
7. For spot quantitation and building expression profiles, the consensus spot pattern is applied to all gel images of the experiment (Fig. 2). The image transformation (step 4) assures that all spots of the consensus pattern arrive at their correct position. A remodeling step makes sure that the predetermined spot boundaries from the consensus are adapted to the real gray levels observed on the target image. All boundaries of the consensus pattern can be found on every gel.
8. Expression profile analysis identifies interesting spots which will be marked for further analysis, protein identification, and interpretation.

## Protein staining

**Fig. 3:**
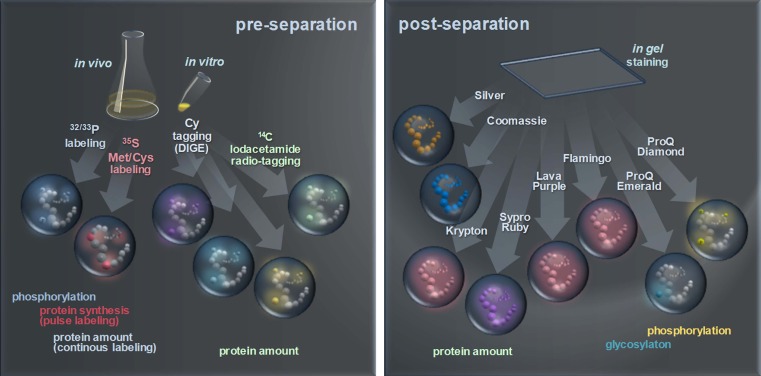
Protein labeling, staining, and tagging techniques for the selective detection of proteins. By multiplexing detection approaches, image analysis may relate different subsets of the proteome such as phosphorylated or glycosylated proteins

Various techniques are available to make the separated spots detectable (Fig. 3). A direct method that suggests itself is the detection of the UV-induced autofluorescence of proteins (Roegener et al. 2003). Unfortunately, it is still not available for routine analysis, although it generates promising results.

Ideally, a dye should bind noncovalently to the protein after a linear response curve. It should allow for a detection of very low protein amounts because protein concentrations in biological systems may vary by six or more orders of magnitude (Corthals et al. 2000). At the same time, saturation effects have to be avoided because they impede normalized quantitation. In practice, depending on the stain that was used, a 2-D gel image analysis may give quantitative or only qualitative results for most or only a subfraction of the most intense spots. Common approaches for the detection of protein amounts use dyes that ideally bind to proteins much stronger than to the gel matrix or other compounds accompanying the 2-D electrophoresis process.

Most fluorescent dyes are more sensitive than quantitative visible stains (Table 2). Like most absorbing dyes, fluorescent stains give a measure of the accumulated amount of proteins within the sample. There are ruthenium-based dyes (ASCQ_Ru) available that covalently bind to the already separated proteins (Tokarski et al. 2006). They also give a measure of the amount of protein, and they can be used like noncovalently binding fluorescent dyes, but after 2-D separation.

**Table 2:** The most commonly used dyes in 2-D gels

Dye	Principle	Sensitivity	Quantitation	Amount/signal
Coomassie Brilliant Blue	Absorption	Very low	After calibration	Nonlinear
Colloidal Coomassie Blue	Absorption	(very) high	After calibration	Nonlinear
Silver Staining	Absorption	Very high	Impossible	Logistic
Sypro Ruby	Fluorescence	High	Yes	Linear
Ruthenium II tris (bathophenanthroline disulfonate)	Fluorescence	High	Yes	Linear
Flamingo	Fluorescence	High	Yes	Linear
Lava Purple	Fluorescence	High	Yes	Linear
Krypton	Fluorescence	Very high	Yes	Linear

The most striking advantage of applying covalently binding dyes before separation is the possibility to run multiple samples in one gel. Provided that the dyes induce the same positional shift in a protein’s position, samples separated in parallel will give exactly congruent 2-D patterns. As long as the number of samples equals the number of available dyes, 2-D patterns with perfect positional identity can be generated. Difference gel electrophoresis (DIGE) was the first approach that used such a sample multiplexing in Unlü et al. (1998) by simultaneously separating samples labeled with Cy2, Cy3, and Cy5 within the same gel. If the amount of samples exceeds the number of available dyes (3 dyes = max 3 samples per gel), more than one gel have to be used, giving rise to positional variation between gels just like in the traditional setups. Dye multiplexing allows for a quantitative normalization over several gels by using an internal standard, i.e., a mixture of equal aliquots of every sample under analysis (Alban et al. 2003). The internal standard is separated together with Cy3/Cy5-labeled sample pairs on every gel and serves as quantitative reference.

Protein stainings give a measurement of the current protein amount. They are well suited for knock out experiments or analyses of long-term experiments in which treatment or stimulus effects have time to manifest themselves as observable changes in protein levels. Staining techniques that measure the accumulated quantity of proteins can overlook minor changes in protein quantities because they disappear within noise or systematic errors. CVs between 25 and 45% for the same spot/same sample separated on different gels were reported by different authors (e.g., Nishihara and Champion 2002; Eravci et al. 2007). This means that a 1.3-fold change of a protein species cannot be reliably detected. That is why for short-term stimulus response experiments, in vivo labeling techniques can give a more detailed picture of a changing proteome.

## Characterizing specific protein properties

There is a broad range of techniques available that are able to detect specific protein features (Fig. 3), such as phosphorylation. The general approach is to combine a conventional staining method that displays all separated proteins with a specialized stain that highlights protein features, giving separate images that can be recombined using software. The Diamond ProQ (Steinberg et al. 2003) and Emerald ProQ (Patton and Beechem 2002) stains bind specifically but noncovalently to phosphorylated and glycosylated proteins, respectively. Multiplexing the phosphoprotein or glycoprotein pattern with the total protein clearly indicates the modified protein species. For a quantitative analysis of phosphoproteins in a bacterial system, see Eymann et al. (2007). For the redox state detection of proteins, two assays for oxidized thiols based on a radiolabeled tag (Leichert and Jakob 2004) or a fluorescent tag (Hochgräfe et al. 2005) have been suggested. In addition to visualizing the protein’s thiol state, image multiplexing allows the calculation of the degree of oxidation. An assay for the detection of carbonyl groups which are an indicator for irreversible protein oxidation has been successfully applied by Dukan and Nyström (1999) or Mosterz and Hecker (2003). This approach uses immunodetection of 2,4-dinitrophenylhydrazone derivated carbonyl groups.

**Fig. 4:**
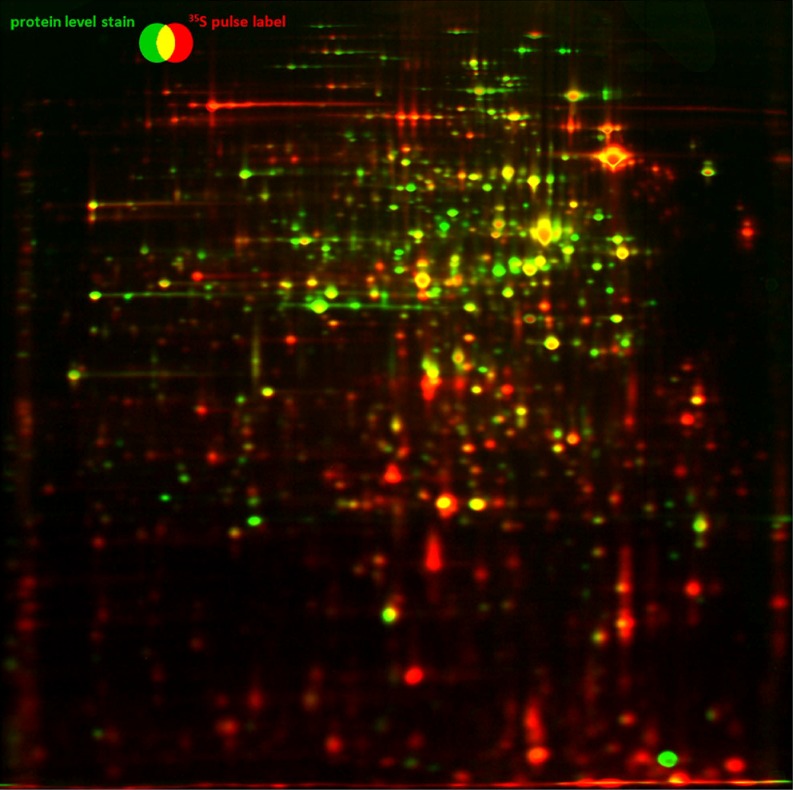
Protein amount (*green*) and protein synthesis (*red*) in a heat shock experiment of *Bacillus subtilis* 168. The synthesis patterns can differ dramatically between different stimuli but can be easily related using the protein amount patterns

Protein synthesis at a given point in time can be detected by using in vivo labeling with radioisotopes. Radiolabeling is perfectly suitable for the detection of changes in protein synthesis during stimulus response studies. Especially ^35^S radioactively labeled amino acids like methionine or cysteine or ^14^C-labeled compounds are applicable. After 2-D separation, the incorporated material is analyzed by phosphorimaging (Amemiya and Miyahara 1988; Johnston et al. 1990). This delivers data with a linear correlation between radioactivity and measured signal over nearly five orders of magnitude. The striking advantage of radiolabeling in stimulus/response experiments is the ability to detect fast but relatively small changes in protein quantities (Bernhardt et al. 1999; Fig. 4).

The spot patterns may be very different when only special protein subsets are imaged, so they are hard to compare. Fortunately, the combination of autoradiography with total protein staining can help to solve this problem. Autoradiograph and densito-/fluorograph from the same gel can be aligned very easily (Fig. 4) because, normally, only minor gel distortions can occur due to staining and washing steps. Images with the total protein amount may then help in finding correspondences between gels because total protein patterns change only to a small extent, so they can be easily aligned.

Protein degradation has been measured by using in vivo pulse chase radiolabeling as well (Kock et al. 2004). The degradation of a radiolabeled subfraction of cellular proteins is observed in a time course experiment by using a series of 2-D gels and looking for proteins whose signals disappear with time.

**Fig. 5:**
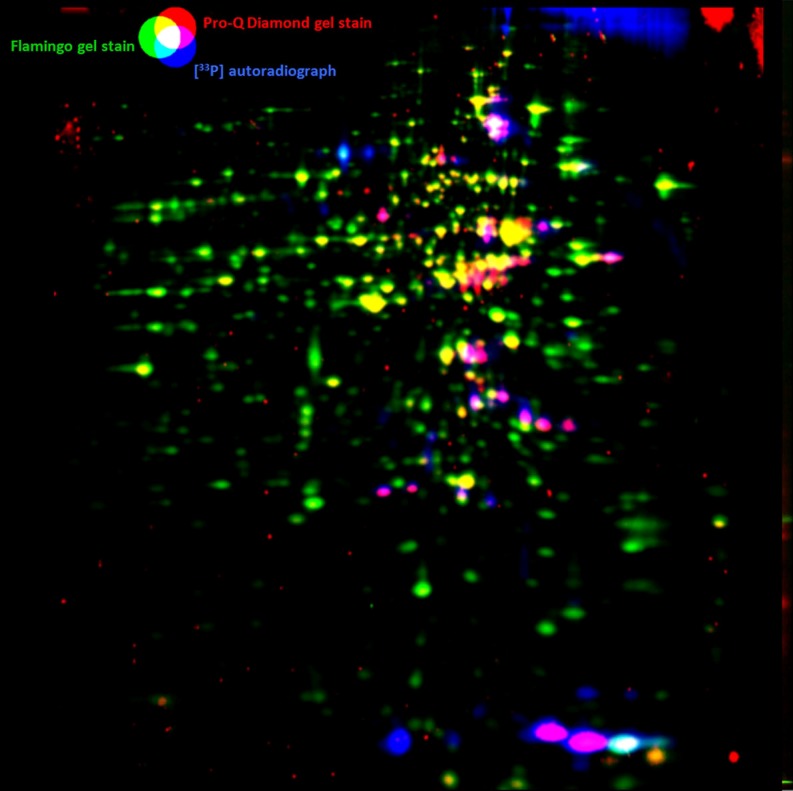
Flamingo-stained protein amount (*green*), Diamond ProQ Phosphoprotein staining (*red*), and 33P in vivo phosphoprotein labeling (*blue*) in an exponentially growing *B. subtilis* 168 sample. While the green and blue subimages seem to be almost complementary, the red subimage highlights spots from the protein level pattern as well as from the phosphate autoradiograph, so it can be used to find correspondences

Radiophosphate ^32/33^P labeling can be used for in vivo detection of short-time effects in protein phosphorylation in the cell. Eymann et al. 2007 suggested to support the analysis with a Diamond ProQ (Invitrogen) stained pattern. The Diamond ProQ stain binds highly specific to phosphorylated proteins but also to a lower extent to nonphosphorylated ones. This allows for the determination of landmarks between stained and radiophosphate-labeled protein patterns that can be used to superimpose ^33^P-labeled and stained protein patterns (Fig. 5).

2-D Western blots are used for the detection of immunogenic proteins as suggested by Haas et al. 2002. Western blots are also suitable for finding modified forms of known proteins (using protein-specific antibodies) or for the detection of protein features (phosphoaminoacid antibodies). Again, superimposing the stained gel and the Western blot can highlight the relevant spots. For a more detailed overview on protein detection techniques, see Patton (2002).

## Recording and preparation of raw image data

Digital images of 2-D gels are acquired using scanners or CCD-camera-based systems. Scanners produce an image by moving a light source and sensor element with CCDs over the 2-D gel. The resolution of the final image is defined by the density of sensors within the CCD element, the speed, and the frequency of the measurements. In CCD-camera-based systems, the 2-D gel is projected onto a CCD sensor array through a photographic lens so the 2-D gel is measured as a whole. Because the photographic lens is an optical system based on refraction, removal of distortions is part of the system’s image preparation. Techniques compensating for lightfield variations are often applied because the central CCD sensors collect more light than those at the borders. The number of single CCD elements on the camera chip determines the resolution of the final gel image. Hybrid systems aim to combine the speed of a camera with the resolution of a scanner by moving a camera over the 2-D gel to produce image tiles that are later assembled using image processing. The challenge is to accurately remove illumination differences and distortions caused by the photographic lens to avoid discontinuities in adjacent tiles. Some scanners have the ability to measure white light, fluorescence, and radiation within one device, as well as simultaneous scanning for different wavelengths. Scanners usually provide higher resolution than CCD cameras while consuming more processing time per image. For technical information, consult Miura (2003) and Tan et al. (2007).

The general rule in 2-D gel image analysis is that the quality of the raw data has a significant impact on the final result. Therefore, it is essential to avoid experimentally caused artifacts and to configure the scanning devices in the best possible way. Background, artifacts, and noise influence the spot detection and quantitation process. Gel disruptions may truncate spots, speckles may mislead the spot detection or distort quantitation, noise can cover low intensity spots, background increases quantity and reduces dynamic range, etc. Background may be caused by insufficiently erased imaging plates (phosphorimaging), insufficient destaining, fluorescing glass plates, gel coverings, and backings. Furthermore, misusing optical filters for fluorescence imaging may cause background. Noise can be produced by high photo-multiplier tube voltages, which leads to the amplification of random signals. Phosphor screens that have not been used for a longer time accumulate noise.

Many software packages allow for postscan image manipulations. One has to distinguish between image manipulations that do not change the quantitative information and those that do, incurring some loss of data in the process. All operations that leave pixels intact do not change the measured data, e.g., rotations in 90° steps, mirrorings, and cropping (removing areas from the images that do not contain information of interest). Linear enhancements of resolution and gray levels can be undone without data loss and do not influence quantitative data because normalization is used in spot quantitation. On the other hand, many operations that are used for image enhancement cause minor changes in spot detection and spot quantitation and should be avoided if possible, e.g., free rotations or free scaling change gray level distribution of the manipulated image.

**Fig. 6:**
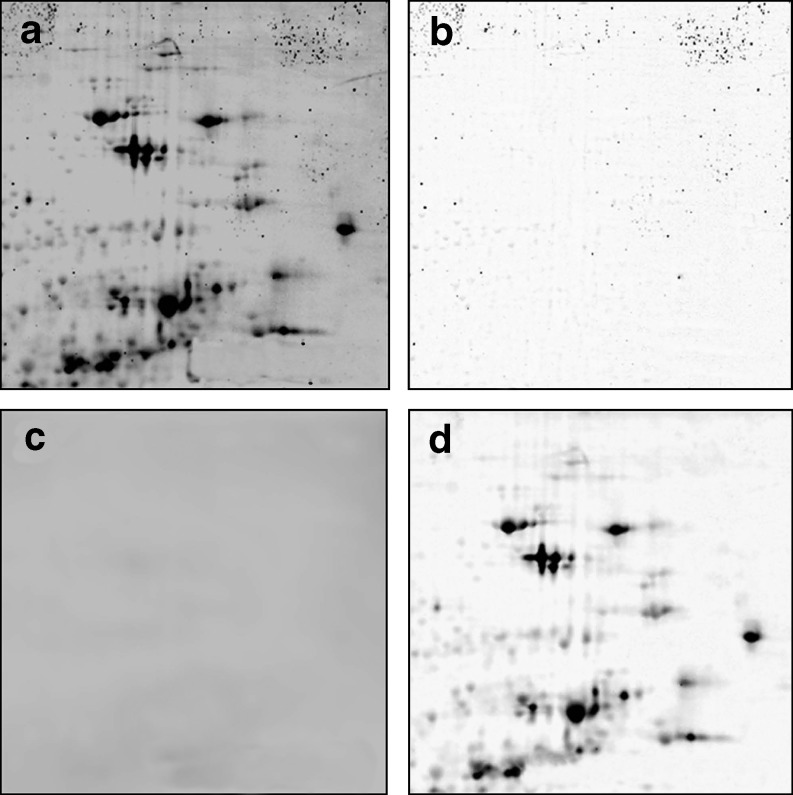
Decomposition of the raw image into background, noise, and cleaned images. Image filters can be used to determine background and noise, leaving the quantitative protein spot information in the cleaned image. **a** Raw image, **b** speckles and noise, **c** background, **d** cleaned image

Image warping leads to changed quantitative data, so quantitation should be done on the original images, or the warping should incorporate a factor for volume compensation (Dowsey et al. 2006) to minimize quantitative side effects. Several types of image filtering algorithms are used by 2-D gel image analysis software to remove background and noise (Fig. 6). These filters are applied within the software for correct quantitation and for optimizing the appearance of the image on the computer screen.

Finally, there are operations that should definitely be avoided because they result in data loss: for example, gamma correction changes the gray levels nonlinearly, blurring, and converting to JPEG format loose data, etc. Another hazard lies in the application of general purpose image manipulation software to special purpose file formats. In the process of, for example, cropping a gel file in Photoshop, essential calibration information can be lost in the resulting file. Therefore, it is advisable to use specialized software (e.g., the software that came with the scanner, or a 2-D gel image analysis program) that understands the characteristics of the file format.

### Removing variations in spot positions—warping

**Fig. 7:**
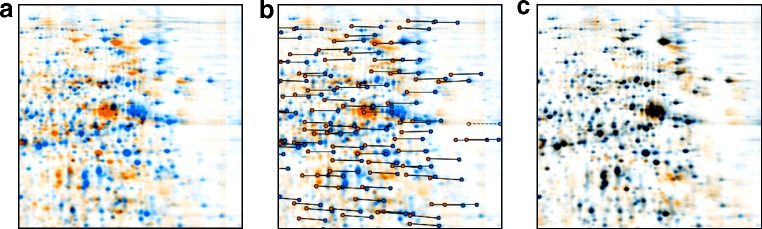
2-D gel image registration by warping. Two images are combined pixel by pixel using a false color display (**a**). Vectors connecting corresponding points (*spots*) on both images are determined automatically (**b**). Transforming the image geometry (warping) according to the vectors produces an exact overlay (**c**). Corresponding spots (*black color*) as well as differences in spot patterns can be easily identified. Data about differences in spot position are used in later image analysis steps (image fusion, transfer of consensus spot pattern)

Unfortunately, the position of a protein on a 2-D gel fluctuates from separation to separation. Even a very experienced experimenter will not be able to produce “perfect” gels whose spot patterns show exact congruency (Fig. 7a). Reasons for changing spot positions may be variations in the pH value of the running buffer, problems of incomplete polymerization of the gel matrix, current leakage (Gustafsson et al. 2002), air bubbles in the gel, or highly abundant proteins that may influence the pH gradient in the IPG gel by their own locally concentrated buffer capacity. Some of these problems can be mitigated by using the DIGE setup or similar techniques (see below) that let multiple samples comigrate on the same gel. However, differing spot positions will still occur in any nontrivial experiment that includes more than one gel. Differences in spot positions are a major challenge in image processing because they impede accurate spot matching and thus the construction of expression profiles.

It turns out that variations in spot positions are localized, i.e., spots that are close together on the gels will have similar gel-to-gel variations in their position (Fig. 7b). This suggests the possibility of eliminating the positional variations by applying an image processing technique called warping. In a more general context, the image processing task is known as image registration; it is, for example, used to combine satellite images from the same region that were taken at different times and angles. In a sense, image registration compensates for variations in the lab process that could not be controlled otherwise. During image registration, similar regions or corresponding spots are searched on both gels in a more or less automated way. This results in pairs of landmark coordinates that link corresponding regions of an image pair to each other. The actual transformation of one image to fit another is not linear (i.e., no simple rotation or scaling suffices), and it can vary considerably between regions. It is important to note that automatic image registration uses the entire available image information and can be done independently of spot detection. Artifacts like gel disruptions, finger prints, and speckles may disturb the finding of the correct warp transform and should be experimentally avoided or removed by the analysis software. Image registration techniques have been introduced by Compugen’s Z3 and DECODON’s Delta2D in 2000 and are meanwhile adopted by other software packages. For surveys of image registration as applied to 2-D gels, see Aittokallio et al. (2005) and Dowsey et al. (2003).

Once suitable warpings between gel pairs have been produced and checked using, e.g., dual channel images (Fig. 7b,c), they can be combined to allow for the registration of every gel in the experiment to every other gel. By knowing the necessary transforms between the images, the software can essentially remove all differences in spot positions as needed for, e.g., precise spot matching or fusion images (see below). In rare cases, no suitable warp transform can be found, e.g., if spots switched their relative positions or if the patterns are so different that no obvious landmark pairs can be found (e.g., stimulus/in vivo labeling experiments, dye multiplex experiments). Under these circumstances, alternative experimental setups should be used. One example is the linking of gel images via comigration of differently labeled samples in the same gel (DIGE) or by using helper gels containing mixtures of two samples A and B to find an implicit warp transform between two gel images via a helper image (Eymann et al. 2007).

## Spot detection and quantitation

**Fig. 8:**
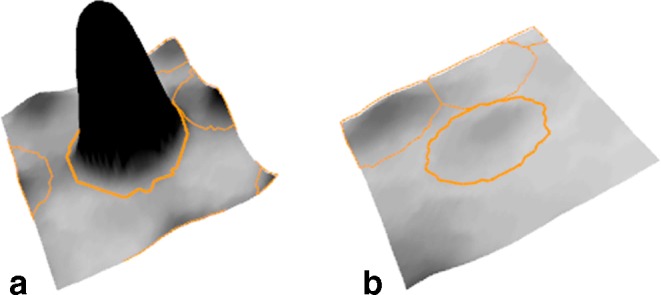
Spot boundaries for high (**a**) and low abundance (**b**) spots

**Fig. 9:**
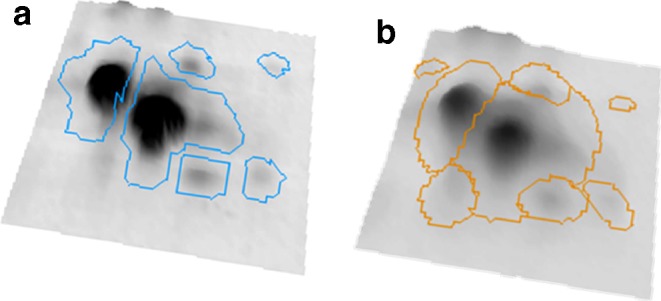
Spot boundaries produced by segmentation (**a**) and subsequent modeling (**b**)

The goals of this step are to find the spot positions, find their surrounding boundary, and determine their quantities. There are two basic approaches that are used in current software: image segmentation and model-based quantitation. The segmentation approach partitions the image into nonoverlapping segments, essentially classifying each pixel as belonging to a certain spot, or as being part of the background between spots. Spot boundaries and quantities are then derived from the spot’s pixels. The segmentation of the image can take various characteristics of the image into account: raw intensity, slope, and classification of pixels in the surrounding region. The advantage of this approach is that the image is clearly separated into spots and “nonspot” areas which are easy to assess by a user. If the software allows editing of spot boundaries, then any desired spot shape can, in principle, be obtained. Model-based approaches try to model a spot’s intensity as a Gaussian normal distribution or some variant thereof. A spot’s quantity and boundaries are then derived from the model (Fig. 8). The use of a Gaussian is motivated by the “3-D shape” of spots (Fig. 8) and by general considerations on diffusion processes in the gel. Model-based approaches limit the range of possible spot shapes, thus leading to more “natural” outlines. On the other hand, irregular spot shapes are poorly represented by simple models. Spot models can be used in the subsequent quantitation, with overlapping spots being represented as the sum of multiple single-spot models. Delta2D offers a hybrid between segmentation and modeling: starting with segmentation, spots are modeled as Gaussians, and their nonoverlapping boundaries are derived from the models (Fig. 9).

The particulars of the methods used for spot detection are often proprietary information of the software vendors, and thus not publicly available. For a survey of published work, see Dowsey et al. (2003).

The spot detection process can be controlled by setting software-specific parameters, such as expected size of a spot in pixels, or even expected number of spots. Due to ambiguities in the gel images (merged spots, weak spots, noise), automated spot detection can only be a heuristic process in some areas. The user will, therefore, sometimes want to change the spot pattern by removing spots, splitting spot clusters, or joining spots. Manual intervention has a downside as well: Individual users have different perceptions about the “correct” spot shapes, so reproducibility between different operators of the software suffers. It is, therefore, advisable to reduce the necessary manual interventions to a minimum, e.g., by defining points as “markers” for the creation of new/splitting of existing spots and letting the software determine the adapted boundaries.

**Fig. 10:**
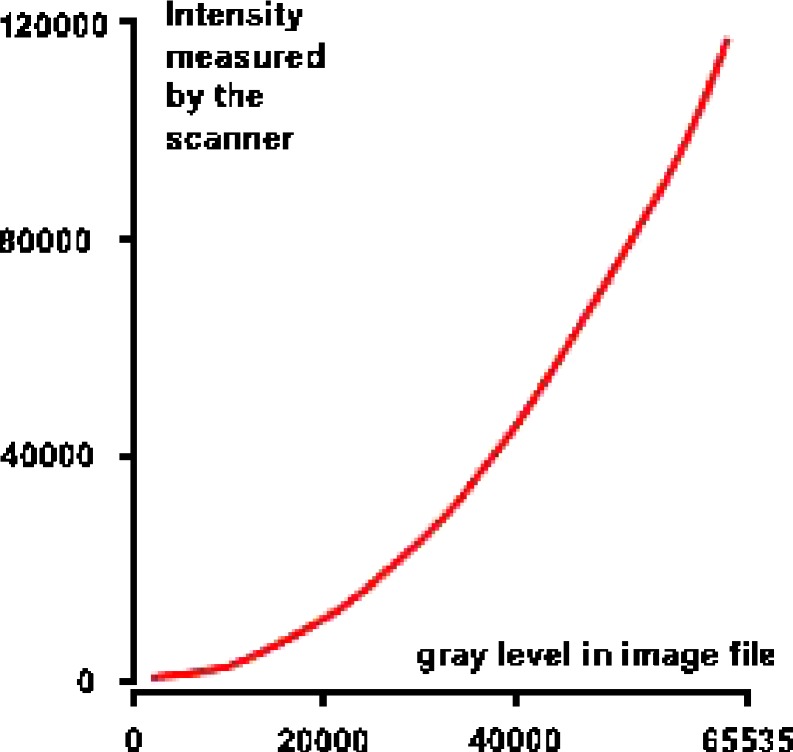
Example of a gray level calibration curve that is used in special image file formats. Gray levels found in the image file have to be interpreted according to the curve before being summed up for quantitation. The curve has lower slope in the low intensity range resulting in better quantitative resolution for weak signals

In the simplest case, one assumes that gray values found in the image file are directly proportional to image intensities and, by extension, protein quantity in the small gel area corresponding to the pixel. However, more advanced imaging equipment utilizes calibration information that should be used to arrive at correct quantities:

Calibration inherent in the file format (Fig. 10). Some imaging devices can measure more intensity values than what fit into the available image file formats. For example, if the imaging device can measure a range of 120,000 intensity values, this range cannot be stored into a 16-bit TIFF image because the file format only provides 65,536 possible gray levels per pixel. One way to deal with this is to transform the measured intensity values linearly into the range of gray levels in the image file. Alternatively, because, for lower signal intensities, higher accuracy is desirable, a nonlinear function (e.g., square root transform as illustrated in Fig. 10) is applied by some imaging devices. This provides a more precise representation of lower-intensity pixels at the price of lower accuracy for high intensities. The corresponding vendor-specific file formats, e.g., Fuji’s IMG/INF format (Fujifilm, Düsseldorf, Germany) and the GEL format used in devices by Molecular Dynamics (Sunnyvale, CA, USA) and GE Healthcare (Munich, Germany), have to be interpreted accordingly by the analysis software.

Calibration of the device. Another type of calibration may have to be applied to eliminate variance between imaging devices of the same model. While most of the laser scanners in the market have a built-in autocalibration, many flatbed scanners need to be calibrated manually. This can be done by using calibration wedges which are offered by several resellers, e.g., Stouffer Industries (Mishawaka, IN, USA), Danes-Picta (Praha, Czech Republic), UVP (Upland, CA, USA). Secondary calibration can be applied if the user knows about the relationship of protein amount and measured signal.

Finally, one can take into account the dye-specific response curve for protein staining. Protein concentration wedges may help to find a fluorescent or absorptive stain-specific transfer function that may help to derive the protein amount from the emitted light or the measured absorption, respectively. It is to be expected that even different protein species have different response curves resulting from their biochemical properties.

**Fig. 11:**
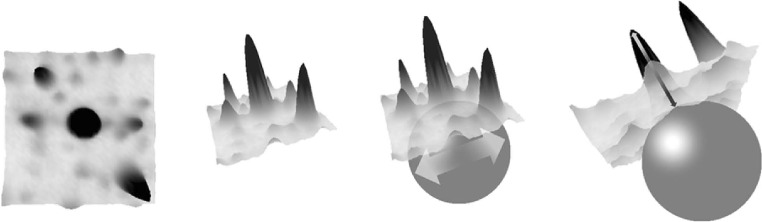
Background subtraction using the rolling ball approach

Image background is generated by material that is stained but not part of a protein spot. The method of background subtraction can have a significant influence on spot quantities; therefore, it is important that background quantities are made explicit by the software instead of being silently subtracted from a spot’s quantity. Background levels can vary considerably between regions on a gel and between gels. Some background subtraction methods are based on the gray levels at the spot boundaries, other approaches are based on the entire available image data. One example for a background estimation that is based on the spot boundary is DeCyder’s (GE Healthcare) rule: Background is determined by the tenth percentile value of all intensity values on the boundary. A background model based on local minima was used by Tyson et al. (1986). The Melanie II software (Appel et al. 1997) calculated background based on a polynomial that is fitted to image intensities. A related approach is the rolling ball method (Skolnick 1986) that determines background levels by fitting a sphere into the 3-D “landscape” of the image (see Fig. 11). The sphere needs to be large enough such that the ball will not go too deep into the spots. Background levels are then determined relative to the center of the ball when it touches the image surface.

## Normalization of spot quantities

**Fig. 12:**
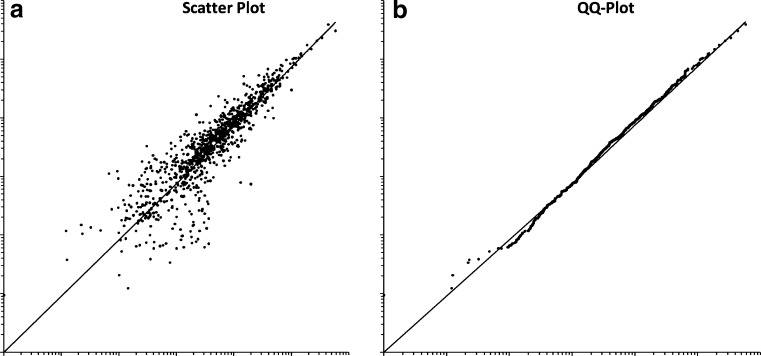
Scatter plot (**a**) of logarithmic spot quantities on two gels from different samples. Spots were normalized based on total spot quantity. **b** The quantile–quantile plot (*QQ plot*) of the same data. Spots are sorted by quantity separately on each gel; spots of corresponding ranks are plotted. The QQ plot makes it easier to compare the spot volume distributions; in an ideal experiment, all points would lie on the diagonal line. The diagram shows that the quantity distributions on both gels are nearly equal, indicating a successful normalization

Normalization procedures aim to mitigate systematic differences between images. Such variation can occur in protein loading, imaging exposure times, and dye/staining efficiency. Normalization starts with raw spot volumes and relates them to spot volumes on the same or on other gels. The simplest common procedure is to normalize for total volumes. The corresponding normalization rule is to set the total spot quantity to 100% on each gel image. For every spot, its proportion of total quantity is then computed. This rule results from the assumption that there is the same total protein amount on all gels. With this procedure, errors such as different protein loads, differences in staining times, scanner exposure time, or detector sensitivity can be compensated. For some instances, the total spot intensity is distorted by one or a few very strong spots leading to a skewing in the scatter plot (Fig. 12a). A countermeasure is to exclude some of the strongest spots (e.g., the 10% strongest spots) from the calculation of total spot intensity, i.e., removal of outliers.

A more general approach is to postulate that the spot intensity distributions (or some of the distribution’s parameters) are equal between gels/gel images. Therefore, e.g., DeCyder (GE Healthcare 2007) tries to match the distributions by adapting parameters of a normal distribution. Another approach is to use only a subset for normalization, e.g., by declaring that certain spots belong to “housekeeping” genes. It is hard to justify a subset like this, given that their being unaffected by the phenomenon under study is just a hypothesis. In principle, one could also spike the gel with a defined mixture of proteins with known quantities. We are, however, not aware of any published work that used this approach. More advanced normalization methods that were originally developed for the analysis of microarray data have been successfully applied in the context of 2-D gel analysis (Fodor et al. 2005). Spatial bias, i.e., a systematic increase or decrease in spot intensities in a gel region, can be observed in experiments and could be corrected by incorporating it in an error model analogous to those used in microarray analysis (Kreil et al. 2004).

The implicit assumption in all normalization procedures is that the majority of proteins are not affected by the phenomenon under study, or the technical variations in the process. Therefore, the global intensity distributions should be equal. The similarity of two distributions can be assessed using a quantile–quantile (QQ) plot (Fig. 12b). It is a two-dimensional plot where spots are compared based on their rank, i.e., the strongest spot on one image is plotted against the strongest spot on another image, etc. For identical distributions, the QQ plot is a diagonal line. Systematic deviations like an overall higher intensity are easily visible.

Normalization procedures that take into account only intensities on one image might be called “vertical” (using columns in an imagined spot intensity table); they modify values based on values in the same column (i.e., image). A different class of normalizations uses intensities from other gels/images; these might be called “horizontal” normalizations. The most prominent example is the DIGE setup using a common internal standard on all gels, usually in the Cy2 channel (Alban et al. 2003). The internal standard is composed by mixing all samples from the experiment in equal amounts. Spot intensities are then normalized by dividing them by the intensity of the corresponding spot on the standard image (Cy2 channel on the same gel). In addition to the gel-to-gel variations, these normalization methods have to cope with differing backgrounds and differing distributions of spot quantities between dyes. The resulting statistics are very similar to the relative expression levels that are produced in competitive hybridization microarray experiments. For a critique of the then-current DIGE normalization used in DeCyder and an alternative normalization method, see Karp et al. (2004).

As a result of the spot detection and quantitation step, the user gets a variety of data on each gel spot, including normalized spot quantity, background, spot outline, position of the spot center, and spot quality measures.

Of course, it would be desirable to relate spot intensities that are obtained from 2-D gel image analysis directly to protein molecule counts in the original sample. However, the relation between original protein quantity in the sample and measured spot intensity is influenced by various intervening factors:

1. loss of sample during entry into the IEF gel,
2. efficiency of transfer from first to second dimension,
3. protein loss during staining,
4. staining efficiency,
5. a protein’s staining curve over time,
6. staining curve over concentration, and
7. dye bleaching.

Given the biochemical diversity of protein molecules, it is to be expected that there are some proteins with a nonlinear relation between concentration and intensity. As a result, one expects to obtain only quantitative results of a relative nature, referring to the same protein species, similar to “this protein is two times stronger in sample A than in sample B.” It is essential to track the protein across gels for this relative quantitation to work. Although the limiting factors mentioned above may seem a bit discouraging, recent controlled experiments (Eravci et al. 2007) show that even a 25% change in quantity can be reliably detected for a substantial fraction of the proteins provided that one controls experimental variation and software-related problems that have a diminishing effect on reproducibility (mainly spot matching, see below).

## Building expression profiles

For comparing the spot intensities over a whole experiment, each spot on a certain gel has to be mapped to the corresponding spots on the other gels in a process called spot matching. The quality of the matching depends on the quality and the reproducibility of electrophoretic separation and spot detection as well as on the methods employed by the software. In the ideal analysis, spot matching would produce exactly one expression profile for every protein species that is visible on any gel.

The challenges in spot matching lie in the differences in migration positions between gels, changes in the spot pattern itself (i.e., proteins that are missing or very weak in one of the samples), ambiguities in the gel data (e.g., more or less well-resolved spot clusters). Due to these difficulties, spot matching has been, alongside with spot editing, the most time-consuming step in the analysis when using traditional software. The traditional approach consists of doing separate spot detections for each individual gel image of the experiment. This results in differing spot patterns, even for replicate samples. In a subsequent step, the user has to revise these results and, if necessary, to correct missing or false positive spots. Furthermore, regions that were detected as separate spots on one gel but as a single spot on another have to be split or joined by hand. Unfortunately, these problems grow with the number of gels (Voss and Haberl 2000), severely limiting the statistical benefits that come with larger sample numbers. For example, in a study that used plasma from five different individuals, taken at six time points (Fodor et al. 2005), there were fewer than 150 spots matched across at least 40 of the total 45 DIGE gels, out of 2,385 spots total. Although it seems possible with enough manual work to improve expression profiles, both the inherent ambiguity in the images and the sheer amount of work necessary result in the acceptance of mismatches and gaps in expression profiles with classical packages. Spot-matching-related problems affect the statistical analysis: Gaps in expression profiles have to be treated as missing values. Mismatches are the equivalent of substituting a random value into the expression profile. Both effects decrease the statistical confidence substantially.

To overcome these problems, a different approach to construct expression profiles has been suggested and implemented in recent years. The approach is based on a spot consensus pattern that is derived from all gel images of an experiment and then applied to each gel image of the analysis set (Figs. 1 and 2). The consensus pattern is produced by spot detection on a fused image that essentially contains all spots in the experiments (“union fusion,” Luhn et al. 2003). Image fusion is a method to combine multiple images into a new, synthetic image where each pixel is a function of corresponding pixels in the input images. The resulting image looks like a real gel image, and, more importantly, all spots from the experiment are represented on it. Thus, spots that are present only on a few of the gels can be located in the fused image, and properly separated from those surrounding them. When the detection and editing of the consensus spot pattern is finished, spot boundaries are transferred back to the original images. This transfer uses the transformations that were produced from the warping step and used earlier to create the fused image. The spot sizes and intensities differ from gel to gel, so a spot remodeling step is applied to make the spot boundaries fit to the gray level distributions of the original gel images. The spot quantities are then calculated as usual by summing the pixel intensities within the spot boundaries. Because each gel has the same spot pattern (modified by the warping transform) a matching of the spots is quite easy and results in 100% spot matching and complete expression profiles. For spots that are absent on some gels, the method will nevertheless produce a spot quantity which will be near zero after background is subtracted. In essence, one could describe the spot transfer approach as quantitation of corresponding gel image regions. The resulting expression profiles were shown to be superior to those produced by the classical method (separate spot detections on each gel) both in terms of quality as well as time needed for the analysis (Eravci et al. 2007).

## Analyzing gene expression

Having expression profiles at hand, we can now proceed to address the important biological questions of the phenomenon under study. Overall, the task is to distinguish those proteins that are relevant or interesting from those that are relatively unchanged. It is often the case that 2-D gel studies are exploratory in nature, i.e., that one wants to generate hypotheses and find proteins whose role can later be validated using other methods. An example of this is the discovery of protein biomarkers where one searches proteins that are consistently associated with a phenotype. Another group of applications is concerned with elucidating and interpreting subsets of proteins that are involved in a biological process, e.g., coregulated proteins induced by an external stimulus (Bernhardt et al. 2003). Methods ranging from the classical statistical tests to machine learning are applied to address these questions.

As in all experimental work, the design of the experiment is of crucial importance to its success. It is expedient to ask for statistical advice early to be able to make optimal use of resources and limited amounts of sample material. For all but the most exploratory experiments, one will want to use replicates: biological replicates to address genetic and environmental variability and maybe technical replicates (i.e., multiple separations of the same sample) to address the variability in the gel electrophoresis process itself. General guidelines concerning the use of replicates and statistical analyses accompanying published data have been put forward in Wilkins et al. (2006). Of course, a higher number of replicates increase the number of proteins that can be identified as being relevant with statistical confidence. A recommendation for the simplest possible case (two sample groups) is worked out in Hunt et al. (2005). Karp and Lilley (2005) present a power and cost-benefit analysis for DIGE experiments and DeCyder software that can be used as a model for other experimental setups. The type of replicate affects the statistical model that should be used: When applying statistical tests, technical replicates cannot necessarily be viewed as independent observations, so a more refined statistical analysis, e.g., nested analysis of variance (ANOVA), can give better results (Karp et al. 2005). Pooling of samples should be kept to a minimum because it diminishes (or prevents) the ability to assess biological variation within a sample type. In Molloy et al. (2003), the effects of technical and biological variance are investigated for samples of different types (bacteria, cell lines, primary cultures, human samples). For guidance on experiment design, see also Hunt et al. (2005). Of course, experimental variability is different between laboratories, and the software and analysis method used can have a crucial influence on the outcome’s quality. The studies cited here use separate spot detections on each gel and often bias results by selecting only spots that were matched across a sufficient number of gel images.

At the level of expression profiles, 2-D gel analysis is very similar to the analysis of DNA microarrays, so a lot of the methods applied there can be used with minor adaptations. In fact, some of the work on machine learning on 2-D gel data predates the microarray technology (e.g., Appel et al. 1988). Let us highlight the main differences between 2-D gel data and microarray data before we proceed to describe the methods in detail:

1. Running differences between gels add a source of errors for spot matching, whereas in microarray data, matching is trivial because every gene is spotted at a known row and column.
2. Spot detection in 2-D gels is much harder because spots may not be distinct.
3. Gene information is not readily available for spots, so it is harder to correlate or cross-validate expression profiles with gene annotations.

The first two characteristics have the greatest impact on the quality of statistical analysis. Running differences and spot shape ambiguities (e.g., distinct spots on one gel vs overlapping spots on another) create spot matching problems that can lead to faulty assembly of expression profiles and gaps (missing values). By using consensus spot patterns (see above) that are transferred onto all images, one obtains consistent expression profiles. Of course, the ambiguities inherent in electrophoresis, e.g., masking of weak spots by strong ones in a nearby position (Pietrogrande et al. 2002; Campostrini et al. 2005), as well as operator-related variance, are still potential sources of errors and variation.

**Fig. 13:**
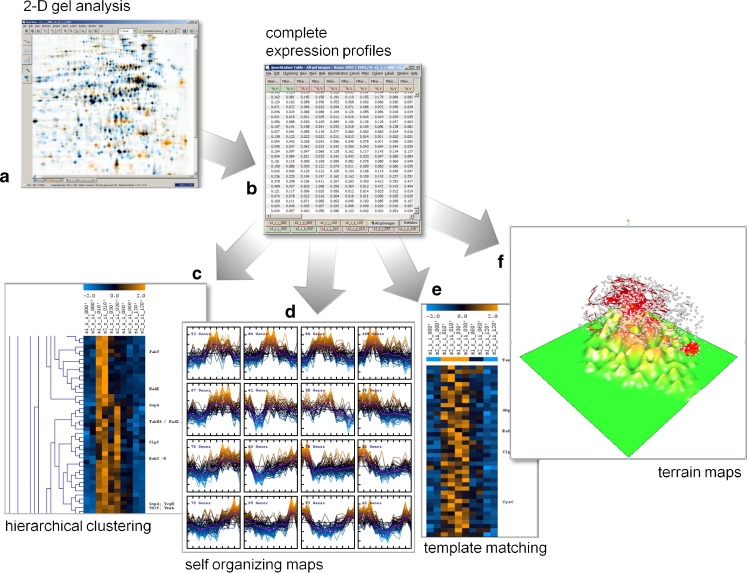
By using a consensus spot pattern in Delta2D (**a**), complete expression profiles (**b**) are generated. Profiles can be imported into DNA array analysis software (here: TIGR MultiExperiment Viewer, TMEV). With appropriate data transformations and normalization, many approaches for data analysis known from DNA arrays can be used for 2-D-gel-based proteome data. Hierarchical clustering (**c**) and self-organizing maps (**d**) group proteins by similarity of their expression profiles. Template matching (**e**) can be used to find proteins that conform to an expression pattern given by the user. Terrain maps (**f**) can give a high level overview of a data set where correlations of protein expression profiles are shown as distances in two dimensions, and protein density is shown in the third dimension (height)

Currently, all 2-D gel analysis software packages come with some basic internal statistical analysis facilities. The advantage of using these facilities as opposed to external programs is that the analysis of expression profiles is tightly integrated with image analysis. For example, it is easy to see a section of all gels around a given spot that was flagged as being differentially expressed. All packages support the export of expression data in tabular form so more advanced methods can be used. Beyond the image analysis packages, there are a few commercial and noncommercial options for the statistical analysis of 2-D gel data. Genedata’s Expressionist (GeneData, Basel) and DeCyder EDA (GE Healthcare) are products that offer multivariate statistics tailored for 2-D gel image data. General-purpose statistics packages like the free and open-source R (www.r-project.org) have extensive facilities for higher-level methods such as principal component analysis (PCA) and clustering. The R-based BioConductor package (www.bioconductor.org) provides access to a wide variety of data analysis methods and graphics facilities that were developed for microarray data. While these command-line-oriented packages offer great flexibility and control as well as some of the latest methods in the field, their learning curve can be steep. A more interactive and visual approach to data analysis is offered by the open-source TIGR Multiple Experiment Viewer MeV (Saeed et al. 2003; http://www.tigr.org/software/tm4/mev.html). MeV combines interactive visualization of microarray data with a wide choice of analysis methods such as hierarchical clustering, self-organizing maps, and PCA (Fig. 13).

### Hypothesis-driven methods

In the simplest case, the experiment is a comparison of two samples A and B (e.g., treated vs control, mutant vs wild type, etc.) The task then is finding those proteins that show significant differences in expression levels. Normally, one will have several replicates per sample, and a statistical test is employed to find differentially expressed proteins. Certainly, the most popular test in this area is Student’s *t* test, where the null hypothesis is that the means of expression levels in samples A and B are the same. Rejecting the null hypothesis then means that the protein under test is differentially expressed. One has to keep in mind that the *t* test makes the assumption that spot quantities within replicates follow a normal distribution which should in principle be tested separately. Additional tests are in use (ProteomWeaver, Bio-Rad, Hercules, CA, USA) that do not depend on the normality assumption (nonparametric tests) like the two-sample Wilcoxon test, also known as the Mann–Whitney *U* test (Conover 2001). In addition to statistical tests, often a fold-change criterion is imposed on the spots. The reasoning behind this is that even true changes in intensity are hard to verify independently if they are small, and that, at least intuitively, a high fold-change in a protein’s intensity is unlikely to be the result of mere chance. For small numbers of replicates, the fold-change criterion is sometimes the only one used.

When applying statistical tests to 2-D gel data, one is faced with the so-called multiple hypothesis testing problem: For each expression profile, a separate test is done. Each test has a certain probability of giving a false positive result, i.e., a protein spot is declared to be differentially expressed, while the difference was due to pure chance. The large number of tests can produce a high number of false positives. For example, in an experiment with 2,000 spots per gel, an accepted false-positive rate alpha of 5% will result in 100 proteins that are found to be “differentially expressed,” although the difference is the result of mere chance. Various procedures try to overcome this problem by adjusting alpha in an adequate way. Bonferroni correction (Weisstein 2007a) controls the probability that there is one single error made in the whole analysis (family-wise error rate), i.e., that there is at least one false-positive protein. However, this method is usually too conservative because, in practice, it is acceptable to have some false positives, depending on the cost of repeating or validating the corresponding results. The proportion of false positives *in the result set* is controlled by the False Discovery Rate (FDR) approach (Benjamini and Hochberg 1995; Pawitan et al. 2005). The false discovery rate is the rate of false positive results among all profiles that were tested positive. While it is difficult to estimate the false discovery rate, the approach in Benjamini and Hochberg (1995) gives a simple procedure to control it, i.e., make sure the false discovery rate is below a given bound. Overall, the FDR approach allows one to strike a balance between the need to find statistically valid proteins of interest and the additional cost that is associated with following up on false positives. For background and applications of FDR and related methods, the reader is advised to consult the reviews in Manly et al. (2004) and Pounds (2006).

For more complicated experiment designs involving multiple factors, ANOVA can be used (Weisstein 2007b; Karp et al. 2005). The basic idea of the method is to find out to what extent the observed variances between different samples can be explained by the experimental parameters, as opposed to biological or technical variation.

### Hypothesis-independent methods

Hypothesis-independent methods were developed for the discovery of patterns in large quantities of possibly high-dimensional data, in the fields of data mining and machine learning. As we expect a small number of fundamental biological processes to be reflected in the expression patterns of a large number of proteins, it makes sense to apply these methods to the analysis of 2-D experiments. Again, much of the work on microarray analysis can be transferred easily because the fundamental unit of data is an expression profile. When using separate spot detections on every gel, the missing values will have to be dealt with by the statistical method, for example, by missing value imputation. A large percentage of missing values decreases the utility of all statistical methods, that is why we recommend using the consensus spot pattern approach described above.

**Fig. 14:**
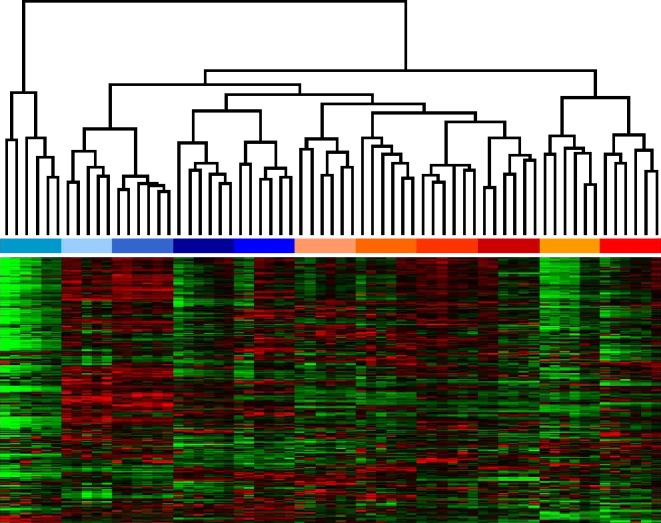
Section of a heat map of a hierarchical clustering of an experiment consisting of 11 individuals with 5 replicate gels each, and 1 average fusion image per individual. Clustering was done for gels (*columns*) and expression profiles (*rows*) simultaneously. Gels are color coded by sample, replicates have the same color, sample A is colored in shades of *blue*, sample B is colored in shades of *red*. The clusterdendrogram for gels shows that replicates were clustered together, and samples are roughly grouped in the higher level clusters. The clustering did not use any sample or replicate information. The *left-most* replicate group is probably an outlier, as it branches off early in the dendrogram. Notice also the cluster structure in the rows, grouping proteins with similar expression profiles (row dendrogram not shown). Expression profiles were generated by spot transfer, hence the absence of missing values. Only about 20% of all expression profiles are shown

Hierarchical clustering refers to a group of methods that aim to group expression profiles or gels by similarity, forming separate clusters that can be further analyzed. Hierarchical clustering of gels can be used to detect outliers and to identify structures in the experiment. Ideally, the cluster composition will reflect the structure of the experiment, e.g., replicates and images from the same sample should end up in the same cluster (Fig. 14). Clustering of images is a good first step in assessing the quality of the quantitative data. Clustering of expression profiles is done to identify proteins with similar behavior, implying that they are coregulated or at least correlated. Again, it is hoped that the cluster structure maps to functional groups or coregulated proteins. The global nature of the cluster display allows for a broad overview and the forming of hypotheses that can then be tested. However, in contrast to the situation in microarray data with 2-D gels, biological annotations of proteins are not available until after protein identification, making it harder to correlate expression behavior to function. The methods and software tools applied in the microarray analysis are applicable here, and the choices a user has to make are essentially the same (Meunier et al. 2007). The first choice is the normalization method, e.g., to standardize expression profiles to be of mean zero and variance one. Then a similarity measure between expression profiles has to be defined, e.g., correlation, or the Euclidean distance. Taken together with further choices such as using single, average, or complete linkage to connect clusters, these combine to create a variety of possible clusterings.

Another method that has been applied with great success in a variety of areas like semantic text analysis (Deerwester et al. 1990) and face recognition (Turk and Pentland 1991) is PCA or the related singular value decomposition. The basic idea is to find a projection from a high-dimensional space into a low-dimensional space (e.g., the plane) such that the structure, especially the variation in the data, is preserved. The principal components are the directions along which the variation is maximal. They can be interpreted as “hidden parameters” of a process or experiment. The tutorial Shlens (2005) provides an accessible introduction, the book chapter Wall et al. (2003) gives background and motivation for microarray analysis.

**Fig. 15:**
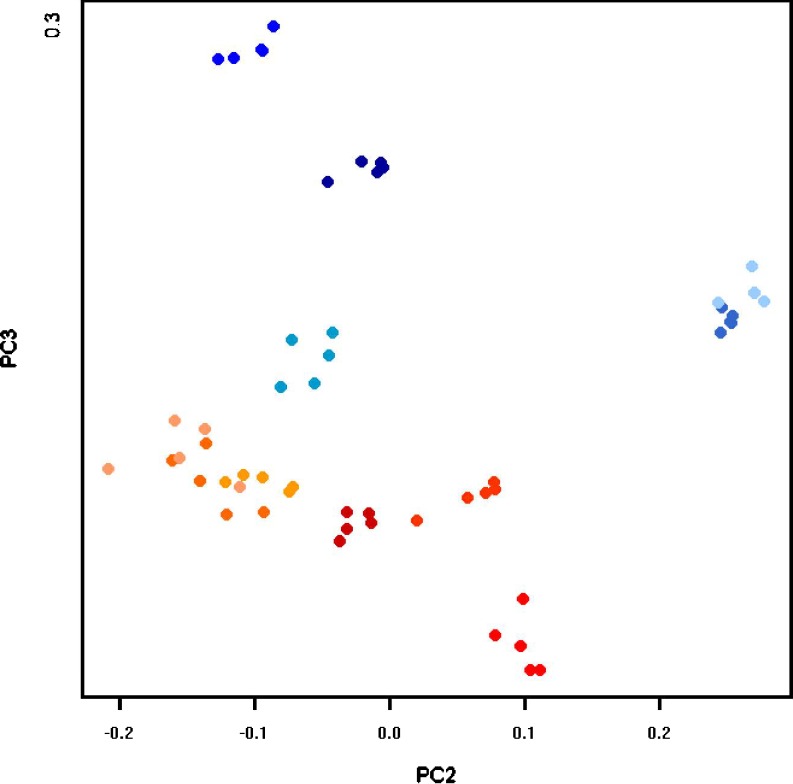
PCA of 54 gels from 11 patients. Gels are color coded according to sample (sample **a**: shades of *blue*; sample **b**: shades of *red*). Notice how replicate gels are grouped closely together. We have chosen the projection onto the second and third principal components because it shows a good separation between samples

As with clustering, PCA can be done for gels or expression profiles. In the first variant, each gel image is considered as a vector with coordinates given by the spot intensities on that gel. For example, an experiment with 24 gels from sample A and 24 gels from sample B and 1,500 spots on each gel would be modeled as a set (or point cloud) of 48 vectors in 1,500-dimensional space. The goal of PCA is then to find a projection of the point cloud in two- or three-dimensional space such that as much as possible the variation of the point cloud is preserved. One hopes that the gels from different samples will be in separate regions of the resulting diagram (Fig. 15). The principal components can then be interpreted as “typical spot patterns” or “eigengels.” Their coordinates can be analyzed to determine which spots are contributing most to the variance, making them candidates for protein identification and biological interpretation.

When PCA is applied to the expression profiles, in our example, we would consider a point cloud of 1,500 vectors (one vector for each expression profile) with 48 dimensions (the expression levels on the 48 gels). The result is a display of the proteins where (hopefully) proteins with close positions are biologically related. Consider a time series experiment, where proteins are switched on and off in stages. If there is a “hidden parameter,” such as a stage in the cell cycle, it will have a systematic influence on the expression levels, and thus increase the variance for the genes taking part in it. This increased variance will then become part of the directions that are used for the projection (the principal components). The principal components were also called “eigengenes,” they can be seen as “typical expression profiles,” see, for example, Alter et al. (2000) and Holter et al. (2000).

## Presentation and visualization: from spots to proteome maps

**Fig. 16:**
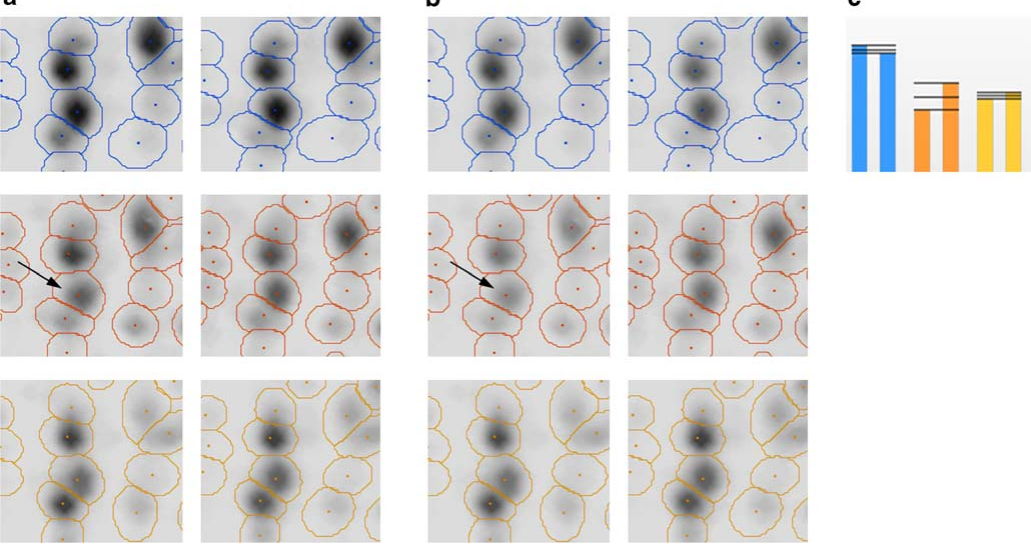
Gel image tiles before (**a**) and after (**b**) multiway histogram equalization. After the equalization, the difference in the highlighted spot (*middle row*, *left* and *right* images) is clearly visible as shown in the expression profile (**c**)

For the presentation of an analysis, as well as throughout the whole process, going back to original images is often useful. Showing whole images or sections thereof in combination with the processed spot data (Fig. 16) is therefore a feature offered by all analysis packages. Let us start with the display of a single gel image. The gray level resolution of current imaging devices (usually 16 bit for 65,536 different values) is much higher than what a computer screen can display. Therefore, the 2-D gel images have to be adapted or enhanced by histogram manipulations that cause a certain loss of information.

The next more complex display is for comparison of a pair of gel images, e.g., treated vs untreated or wild type vs knockout. There are two possibilities: The gels can be shown side by side, or the gels are shown superimposed in a dual channel image (see Fig. 5). For both types, a histogram equalization is necessary to give the user a visual estimate of relative spot quantities. Background can be removed before display, depending on the method that is used for background detection. Even after equalization, the visual impression of spot volumes can be misleading, for example, when comparing a small dark spot with a spot that is less intense but larger. Before gel images are superimposed in false colors, histograms should be equalized, and the chosen colors should be of similar apparent intensity. This makes sure that one image does not dominate the others and negatively influence the estimation of quantities. Ideally, the chosen colors are of similar luminance and located on opposite sides of the color wheel. Triple channel images are sometimes used (red-green-blue channels; see Fig. 6) but difficult to interpret for the untrained eye.

For the display of multiple gels, one often shows tiles containing spots of interest in a side-by-side display. Here, a multiway equalization of histograms is necessary to give suitable visual impressions of spot intensities. Figure 16 shows the effect of multiway equalization on the display of a series of 2-D image tiles. With proper equalization quantity, the visual impression closely corresponds to the normalized quantities shown in the expression profile chart (Fig. 16c).

Presentations of large gel series, for example, for time course experiments, or large projects containing a lot of replicates, will require too much screen space for a side-by-side display. Image warping can help in making an animation of 2-D gel series from time courses by assuring that spot positions do not jump between frames. Bernhardt et al. (2003) used a combination of dual channel images showing protein amount (stained proteins—false colored green) and current protein synthesis (^35^S methionine labeling—false colored red) to monitor the fate of each protein during the development of a bacterial culture along a time line (http://microbio1.biologie.uni-greifswald.de/starv/movie.htm).

Especially for the collection of protein identifications and the presentation of proteome maps, it is very useful to condense spot pattern information from a multitude of gels into a single reference image. This can be done by collecting spot identifications on a single representative gel (Eymann et al. 2004). When using image fusion with the union or max intensity combination function, a proteome map can be generated that shows all proteins that were observed in the experiment. The map shows realistically looking spot shapes and does not ignore differentially expressed spots or dilute rarely occurring spots like it would be the case with average images (Tam le et al. 2006).

A related feature was implemented in Proteomweaver (Bio-Rad) that allows for the combination of different narrow pI-gradients into a global proteome map. This composite map utilizes the much better resolution of narrow pH-gradient strips and supports image analysis as if the data came from a single, very wide gel.

A proteome map normally serves as basis for further, especially physiologically oriented research and is comparable with a DNA array layout. The proteome map defines at which positions a protein spot was identified and can be recovered during gel analysis. A variety of proteome maps of many kinds of samples is available. Most of them show about a thousand different identified protein spots. Additional data can be attached to spots using labels, e.g., protein identification or functional information. Especially in gel regions with a very dense spot pattern, it is a big challenge to display the protein information without obscuring image information with spot labels.

**Fig. 17:**
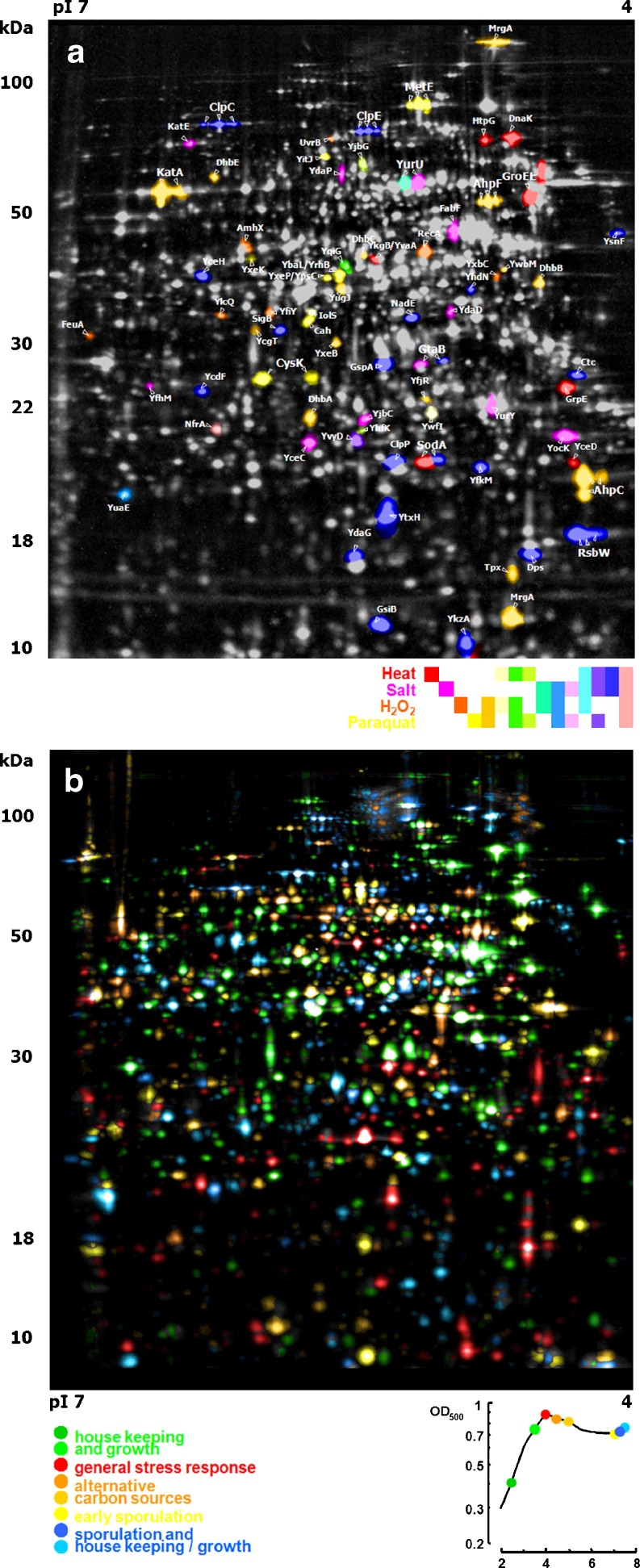
Proteome maps with spot color coding. **a** Stress proteome map of *B. subtilis* 168 (compare Tam le et al. 2006). Spots were color coded according to their induced expression in response to different stress factors. **b** Proteome map of *B. subtilis* 168 in a glucose starvation time course experiment. Spots were color coded according to the growth phase

In proteome maps, color can be used for encoding the association of a protein to a regulatory group (Voigt et al. 2006). In Fig. 17a, proteins above a defined induction factor were grouped by showing them in the same color. If several induction groups are displayed in parallel, Venn diagrams for defining the protein set’s color are used. The presented stress proteome map shows 15 combinations of expression behavior in response to four analyzed stimuli. In general, color coding of protein subsets can be applied for any way of allocating spots to (possibly overlapping) categories.

Color coding also works for highlighting proteins that show elevated expression levels at certain points in a time course experiment. Also here, the information of a time-course-associated gel series is condensed in a proteome map. For each time point, a color is defined in the proteome map, which is then assigned to spots that have their maximal expression level at that time point (Fig. 17b).

In contrast to a tabular display of spot quantities, a proteome map retains that spatial relations between spots as well as typical spot forms. It is, thus, easier to relate visually to newly produced gel images. The color coding of spots allows for easy visual identification of interesting subsets of the proteome.

## Conclusions

Advances in software, algorithms, and experimental methods have kept 2-D gels competitive with and complementary to other methods for proteome analysis. While still not fully automatic, the software-based analysis is not the time-consuming bottleneck in a proteomics experiment anymore. With complete expression profiles, one is able to take full advantage of statistical methods (e.g., hierarchical clustering, PCA) that were established in the analysis of DNA microarray data. We see three main directions for future improvements of the available software: Firstly, the basic image processing algorithms, i.e., spot detection and image registration (warping), should be improved to a point where the need for a human operator is limited to checking the results. Ongoing research in automated registration and the potential for even greater processing power with grid computing (Dowsey et al. 2006) are promising steps towards this goal. The proteomics community can advance the state of the art by freely sharing raw data from a wide variety of experiments, and by establishing benchmarks. Standardization efforts of data formats and reporting requirements are being established for the gel electrophoresis process (MIAPE GEL, currently open for public feedback before publication at http://www.nature.com/nbt/consult); a similar standard for image processing and documentation of results is under development. Both are coordinated by the HUPO Proteomics Standards Initiative (www.psidev.info). The second direction for improvement is defined by the further adoption of advanced statistical methods for the analysis of large 2-D gel experiments. It seems plausible that large numbers of images from multiple studies could be aggregated to give richer “functional profiles” of proteins that show expression levels across a wider range of samples and conditions. The third direction is putting results from 2-D gel analysis into a larger context by combining spot data with functional annotations and data from other experimental techniques. Visualizing the result in biological terms (metabolic pathways, functional categories, etc.) will make it possible to gain new insights from the growing amounts of data accumulated by the “Omics” technologies.
