# Erratum to: The state of the art in the analysis of two-dimensional gel electrophoresis images

**DOI:** 10.1007/s00253-008-1452-z

**Published:** 2008-05-01

**Authors:** Matthias Berth, Frank Michael Moser, Markus Kolbe, Jörg Bernhardt

**Affiliations:** 1 DECODON GmbH, Rathenau-Strasse 49a, 17489 Greifswald, Germany; 2 Institute of Microbiology, Greifswald University, Jahnstrasse 15, 17487 Greifswald, Germany

**Erratum to: Appl Microbiol Biotechnol**

**DOI 10.1007/s10253-007-1128-0**

Due to a technical error, the article “The state of the art in the analysis of two-dimensional gel electrophoresis images” carries an incorrect copyright line. The copyright of the article is with DECODON GmbH, not with Springer-Verlag.”

